# Sparsity‐guided multiple functional connectivity patterns for classification of schizophrenia via convolutional network

**DOI:** 10.1002/hbm.26396

**Published:** 2023-06-15

**Authors:** Renping Yu, Cong Pan, Lingbin Bian, Xuan Fei, Mingming Chen, Dinggang Shen

**Affiliations:** ^1^ School of Electrical and Information Engineering Zhengzhou University Zhengzhou China; ^2^ School of Biomedical Engineering ShanghaiTech University Shanghai China; ^3^ School of Artificial Intelligence and Big Data Henan University of Technology Zhengzhou China; ^4^ Shanghai United Imaging Intelligence Co., Ltd. Shanghai China; ^5^ Shanghai Clinical Research and Trial Center Shanghai China

**Keywords:** convolutional neural network, multiple sparse patterns learning, resting‐state functional MRI, schizophrenia, weighted sparse functional connectivity

## Abstract

The explorations of brain functional connectivity network (FCN) using resting‐state functional magnetic resonance imaging can provide crucial insights into discriminative analysis of neuropsychiatric disorders, such as schizophrenia (SZ). Pearson's correlation (PC) is widely used to construct a densely connected FCN which may overlook some complex interactions of paired regions of interest (ROIs) under confounding effect of other ROIs. Although the method of sparse representation takes into account this issue, it penalizes each edge equally, which often makes the FCN look like a random network. In this paper, we establish a new framework, called convolutional neural network with sparsity‐guided multiple functional connectivity, for SZ classification. The framework consists of two components. (1) The first component constructs a sparse FCN by integrating PC and weighted sparse representation (WSR). The FCN retains the intrinsic correlation between paired ROIs, and eliminates false connection simultaneously, resulting in sparse interactions among multiple ROIs with the confounding effect regressed out. (2) In the second component, we develop a functional connectivity convolution to learn discriminative features for SZ classification from multiple FCNs by mining the joint spatial mapping of FCNs. Finally, an occlusion strategy is employed to explore the contributive regions and connections, to derive the potential biomarkers in identifying associated aberrant connectivity of SZ. The experiments on SZ identification verify the rationality and advantages of our proposed method. This framework also can be used as a diagnostic tool for other neuropsychiatric disorders.

## Graphical abstract

We first propose sparsity‐guided multiple functional connectivity patterns, by integrating Pearson's correlation and connectivity strength‐weighted sparse representation. Then an improved convolutional neural network module is introduced to learn the discriminative features of brain networks with different sparsity and the occlusion method is used to find potential biomarkers related to schizophrenia. The experimental results from the Center of Biomedical Research Excellence database demonstrate the promising performance of our method.

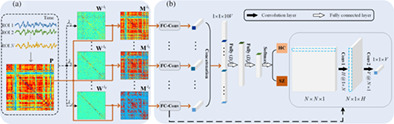

## 1 INTRODUCTION

Schizophrenia (SZ) is a serious chronic disease with unknown etiology (Insel, 2010), and severely affects patient's cognition, emotions (Couture et al., 2006; Tandon et al., 2009), and daily life. It is usually characterized by cognitive distortion, reduced social drive, decreased sociability, and independent living ability. However, SZ diagnosis is complicated due to different symptoms in different individuals, although SZ has led to an international focus on timely diagnosis and earlier intervention.

Resting‐state functional magnetic resonance imaging (rs‐fMRI) is a non‐invasive and highly reproducible brain function imaging method, with no requirement of subjects to perform tasks during the scanning. This avoids the interference caused by the task execution. The rs‐fMRI characterizes local brain spontaneous neural activity by recording blood‐oxygenation‐level‐dependent (BOLD) signals on the time scale (Brown & Eyler, 2006). Brain functional connectivity network (FCN) measures the functional relationship between predefined brain regions of interest (ROIs) based on their BOLD signals, where ROIs are treated as nodes and functional connectivity (FC) between paired nodes refers to the edges (Van Den Heuvel & Pol, 2010). The rs‐fMRI‐based FCNs have been widely applied to diagnosis of neuropsychiatric disease (Chen et al., 2019; Zhang et al., 2022). It is crucial to explore a way to construct the FCN that can preserve information contained in rs‐fMRI, improve the diagnostic accuracy of SZ.

Pearson's correlation (PC) and sparse representation (SR) are commonly used for constructing FCNs. It has been reported in a previous study that the PC‐based methods have relatively high sensitivity for detecting network connections compared with other methods (Smith et al., 2011). PC can measure intrinsic connectivity range from −1 to 1 between paired ROIs, but it cannot measure complex interaction of paired ROIs under the confounding effect of other ROIs. Meanwhile, the PC‐based FCN implicitly assumes that the brain network is densely connected. Emerging evidence has suggested that FCNs constructed considering the influence of multiple ROIs could improve the diagnostic performance of brain diseases (Jie et al., 2016), and the sparsely connected networks are sometimes superior to densely connected ones (Hagmann et al., 2008). The sparse FCNs based on SR have been proposed to characterize the interaction of paired regions under the mutual influence of other ROIs (Lee et al., 2011). In SR, the false connections caused by the low‐frequency spontaneous fluctuations of the BOLD signals and physiological noise are forced to be zero by adding a sparsity prior, and then a sparse FCN can be constructed. Although SR considers the effects of multiple brain regions on the relevance of paired ROIs, the sparse constraint term penalizes each edge equally, which often makes the FCN look like a random network. Compared with PC, the interaction of paired ROIs in the FCN established by SR is weakened, that is, a curtailed amplitude range with SR (Yu et al., 2017, 2019). In addition, most brain network analysis methods usually focus on single construction model, which may ignore the complementary that exists in different brain network construction models. This complementary information may be important for brain disease diagnosis.

With the enormous development of deep learning algorithms, the FC matrix, associated with good grid properties, reveal great compatibility with deep learning methods (Bi et al., 2020; Malkiel et al., 2021). For instance, Zhu et al. introduced an autoencoder network with clinically relevant text information for the diagnosis of mild cognitive impairment, which revealed discriminative brain network characteristics (Ju et al., 2017). Kim et al. (2016) proposed a deep neural network for SZ diagnosis and identification of SZ‐related abnormal FC, with autoencoder pre‐training to initialize weights and $l_{1}$‐norm regularization to control weight sparsity. Bi et al. (2020) designed a convolutional neural network (CNN) combined with extreme learning machine to learn brain network regional‐connectivity features for Alzheimer's disease diagnosis. Meszlényi et al. (2017) proposed a connectome‐CNN combining information from different FCs for mild cognitive impairment diagnosis, and it has been widely used in connectome‐based classification tasks. However, these methods are poorly interpretable and ignore the sparsity and spatial properties of functional brain network, which may lead to difficulty in identifying abnormal FC and weak classification performance.

Correlation‐based brain networks implicitly assume that ROIs are densely connected, which may not match the real FC properties. Therefore, Li et al. (2021) defined edges by hard thresholding (edges with top 10% connection strength are retained) partial correlations to achieve sparse brain network. Z. Wang et al. (2022) proposed a distribution‐guided network thresholding learning method to adaptively generate an FC‐specific threshold for each connection in an FC network according to the distribution of connection strength between subject groups for brain disease diagnosis. In another recent study (L. Wang et al., 2021), PC and the *k*‐nearest neighbors graph are used to build sparse connectivity graphs where the connectivity strength of the first *k* edges of each node is kept, by which the influence of false connections is reduced. However, these sparsification methods ignore the topology in FC networks with different sparsity. Studies have shown that multi‐sparse learning may be useful for exploring real FC network representations (Jie et al., 2014).

In this paper, we propose an SZ classification framework based on CNN and sparsity‐guided multiple functional connectivity (SMFC). Specifically, a weighted sparse representation (WSR) construction method is introduced to generate multiple FCNs with different sparsities for sparsely guiding the PC matrix. By combining PC and WSR methods, the intrinsic correlation between pairs of ROIs can be preserved and the interactions between multiple ROIs can be considered simultaneously. Furthermore, by considering the spatial properties of FCN, we introduce an FC convolution (FC‐Conv) to learn the functional representation of each sparse FCN, and then combine these feature representations for SZ diagnosis. The contributions are summarized as follows. (1) We propose a new analysis framework (SMFC‐Net) integrating PC and WSR methods, as well as a modified CNN for brain disease diagnosis. (2) We obtain better classification performance compared to state‐of‐the‐art (SOTA) methods and discover the important brain regions in classification, which demonstrates the rationality of our method in SZ diagnosis. (3) The source codes of our method have been released to the public at https://github.com/pancccool/SMFC-Net.

## 2 MATERIALS AND METHODS

### 2.1 Materials

#### 2.1.1 Data acquisition

In this study, the experimental data comes from the Center of Biomedical Research Excellence (COBRE) data set. Detailed information of this data set can be found at http://fcon_1000.projects.nitrc.org/indi/retro/cobre.html. All patients are identified according to DSM‐IV diagnostic criteria by qualified psychiatrists, and symptom severity is assessed using the Positive and Negative Syndrome Scale. Exclusion criteria include presence of other DSM‐IV disorders, history of substance abuse, and clinically significant head trauma. Using DSM‐IV criteria, healthy controls (HCs) are also confirmed to be free of SZ or other mental disorders and not to have a history of substance abuse or clinically significant head trauma. The rs‐fMRI is obtained by single‐shot full *k*‐space echo‐planar imaging sequences with the following parameters: repetition time (TR)/echo time (TE) = 2000/29 ms, acquisition matrix = 64 × 64, slices = 32, voxel size = 3 mm × 3 mm × 4 mm, and number of volumes = 150.

#### 2.1.2 Data preprocessing

The first 10 volumes are discarded to stabilize the rest data. The remaining volumes are preprocessed by DPABI toolbox (Yan et al., 2016) as follows: (1) slice timing correction; (2) motion correction and denoising; (3) the functional scans are spatially normalized to a standard template (Montreal Neurological Institute) and resampled to 3 mm × 3 mm × 3 mm; (4) linear drift, white matter signals, cerebrospinal signals, and head motion parameters based on Friston‐24 regress out from the BOLD signals; (5) spatial smoothing (8 mm FWHM Gaussian kernel); (6) band‐pass filter (0.01–0.08 Hz) reduce low‐frequency drift and high‐frequency physiological noise; (7) the automated anatomical labeling atlas (AAL2; Tzourio‐Mazoyer et al., 2002) is used to parcellate the brain into 120 ROIs (60 per hemisphere). After data preprocessing, the mean time series is extracted for each ROI by averaging signals of all voxels within each region. To facilitate the follow‐up analysis, the extracted time series of each ROI is normalized to zero mean and unit variance.

Subjects (*n* = 147 before screening) are excluded from the study if (1) the average displacement due to head motion during fMRI scanning, as estimated from the realignment parameters, exceeded 0.50 mm (Power et al., 2012); (2) the diagnosis results from the DSM‐IV criteria are unrelated to SZ (*n* = 4 subjects); (3) the data are disenrolled (*n* = 2 subjects); and (4) the data acquisition process is incomplete (*n* = 1 subject). The final remaining subjects included 57 SZs and 68 HCs. The demographic information of the data set is shown in Table 1.

**TABLE 1:** Demographic information of the participants from COBRE data set.

Category	Numbers	Gender (M/F)	Ages	Handedness (L/R/both)
SZ	57	48/9	36.68 ± 13.500	8/48/1
HC	68	46/22	35.04 ± 11.205	1/65/2

*Note*: The ages are denoted as mean ± standard deviation.

Abbreviations: COBRE, Center of Biomedical Research Excellence; L/R/both, left/right/(both left and right); M/F, male/female.

### 2.2 Overview of the framework

**FIGURE 1:**
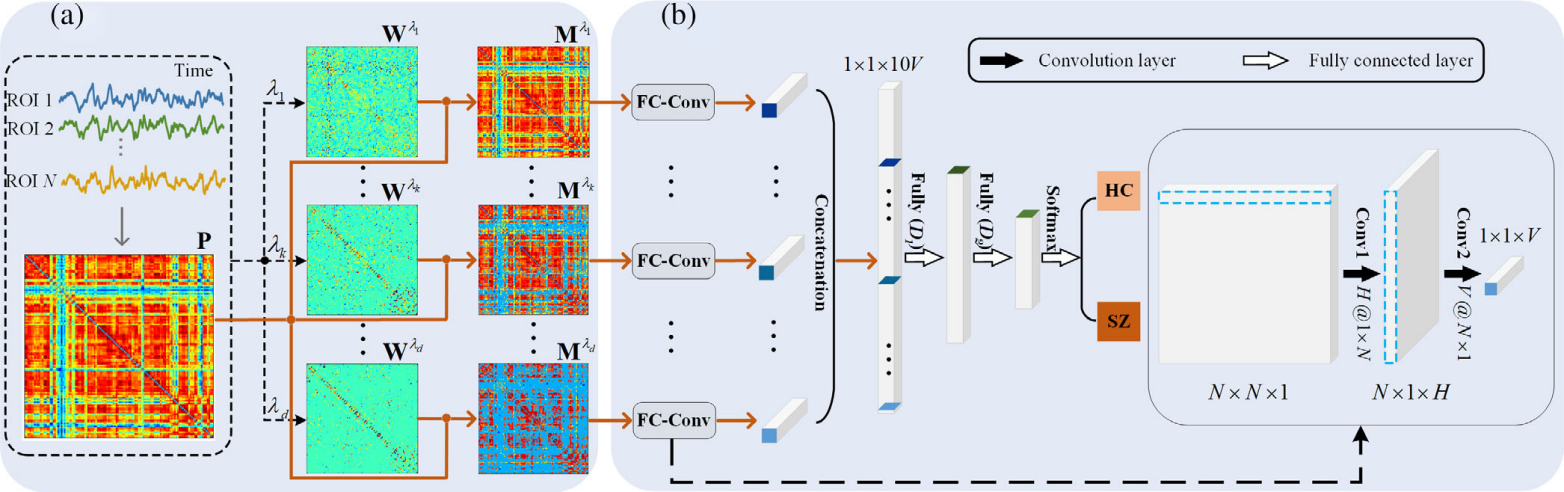
The framework of our proposed SMFC‐Net for SZ classification. (a) The construction process of FCNs. The time series are extracted from *N* ROIs, and a Pearson's correlation matrix **P** is calculated from the time series. $W^{\lambda_{k}}$ is a sparse matrix calculated based on weighted sparse representation, where λ is the regularization parameter and its range is $\left[2^{-4},2^{-3},\cdots ,2^{5}\right]$. $M^{\lambda_{k}}$ is the sparsity‐guided FC matrix. In part (a), the red arrow indicates the combination of **P** and $W^{\lambda_{k}}$ into the sparsity‐guided multiple FCs matrix $M^{\lambda_{k}}$ through sparse guidance, and the black dotted arrow indicates the construction process of $W^{\lambda_{k}}$. (b) The convolutional neural network. Each FC‐Conv contains two convolutional layers, that is, Conv1 (regional connectivity convolution layer) and Conv2 (spatial integration convolution layer). Here, the kernel sizes in two layers are 1×N and N×1 (with the corresponding channel numbers of *H* and *V*), respectively. “Concatenation” denotes that we connect the output (i.e., vector 1×1×V) of all FC‐Convs to obtain 1×1×10V features. Furthermore, the two fully connected layers (with their numbers of units as *D*_1_ and *D*_2_, respectively) and a softmax layer are used for SZ classification. FC‐Conv, functional connectivity convolution; FCN, functional connectivity network; ROI, region of interest; SMFC‐Net, convolutional neural network with sparsity‐guided multiple functional connectivity; SZ, schizophrenia.

We propose to construct a new framework to establish the sparsity‐guided multiple FCs, which combines the advantages of both PC and WSR. Figure 1 shows the schematic illustration of our proposed SMFC‐Net, consisting of two components, that is, (1) one component is the construction of FCNs and (2) another one is a CNN. Specifically, we first compute the PC coefficient matrix **P** to characterize the connectivity strength using the resting‐state BOLD signal. Then, we construct *d* sparsity matrices (i.e., {$W^{\lambda_{k}}$}) based on WSR with weighted sparsity constraint term (Yu et al., 2017) to represent different network sparse topologies (*d* = 10 in our work), where $\lambda_{k}$ represents the sparse prior parameters. $W^{\lambda_{k}}$ are first binarized (0/1) and then applied to **P** to obtain the sparsity‐guided FC matrix $M^{\lambda_{k}}$ as shown in Figure 1a. After that, we establish an FC‐Conv architecture with two convolutional layers as shown in Figure 1b, where the FCNs $M^{\lambda_{k}}$ are the inputs of the FC‐Conv. Finally, the outputs of a battery of FC‐Convs are concatenated, and the two fully connected layers and a softmax layer are adapted for SZ classification.

### 2.3 Convolutional neural network with sparsity‐guided multiple functional connectivity

#### 2.3.1 PC matrix

PC directly measures FC of ROIs by calculating the correlation coefficient of paired ROIs, ranging from −1 to 1. The PC coefficient between two brain regions is calculated by

(1) $$P_{\mathrm{ij}}=x_{i}^{T}x_{j,}$$

where $x_{i}={\left[x_{1i},x_{2i},\cdots ,x_{\mathrm{Li}}\right]}^{T}\in {\mathbb{R}}^{L}$ and $x_{j}={\left[x_{1j},x_{2j},\cdots ,x_{\mathrm{Lj}}\right]}^{T}\in {\mathbb{R}}^{L}$ denote the normalized mean time series of the ⅈth and jth ROIs, respectively. *L* = 140 is the length of the time series. The whole brain PC matrix is $P=X^{T}X\in {\mathbb{R}}^{N\times N}$, where $X=\left[x_{1},x_{2},\cdots ,x_{N}\right]\in {\mathbb{R}}^{L\times N}$ denotes the whole‐brain BOLD signals, and *N* = 120 denotes the number of ROIs. Then the dimension of the PC coefficient matrix **P** is *N* × *N*. The functional brain network measured by PC is supposed to be densely connected. Many methods set an appropriate additional threshold to the correlation matrix for eliminating false connections in a correlation matrix. One of the commonly used methods is to preserve edges with high absolute weights/values by choosing a percentage threshold parameter based on the FC matrix. The sparse FCN constructed in this way still preserves many unwanted false connections, while some important connections are discarded inevitably (Wee et al., 2014).

#### 2.3.2 Network based on WSR

In our previous research (Yu et al., 2017), the WSR is able to characterize interactions of paired ROIs with the interference of other ROIs in the network by combining sparsity and pairwise similarity. The WSR is depicted by a weighted $l_{1}$‐norm, which is formulated by

(2) $$\min_{w_{i}}\frac{1}{2}∥x_{i}-X^{\left(i\right)}w_{i}{∥}_{2}^{2}+\lambda ∥c_{i}⊙w_{i}{∥}_{1},$$

where $w_{i}={\left[W_{1i},W_{2i},\cdots ,W_{\mathrm{Ni}}\right]}^{T}$ denotes a column vector composed of the connecting edges between the *i*th ROI and all the others. $X^{\left(i\right)}=\left[x_{1},x_{2},\cdots ,0,x_{i+1},\cdots ,x_{N}\right]\in {\mathbb{R}}^{L\times N}$ denotes the corresponding dictionary when expressing the BOLD signal $x_{i}$ of the *i*th brain region. The *i*th column of the dictionary is set to 0 to avoid trivial solutions. We denote $c_{i}={\left[C_{1i},C_{2i},\cdots ,C_{\mathrm{Ni}}\right]}^{T}\in {\mathbb{R}}^{N\times 1}$ as a penalty weight vector corresponding to $w_{i}$, and ⊙ as the element‐wise multiplication. The penalty weight $C_{\mathrm{ji}}$ is defined as an inverse proportion function of the pair‐wise correlation $P_{\mathrm{ji}}$, that is, $C_{\mathrm{ji}}=\exp\left(-P_{\mathrm{ji}}^{2}/\sigma \right)$, where σ denotes the parameter to adjust the attenuation speed of the corresponding connectivity‐strength weight empirically set as 0.2 to be same as our previous work on the identification of mild cognitive impairment (Yu et al., 2017, 2019). Thus, a larger correlation $P_{\mathrm{ji}}$ will result in a smaller penalty $C_{\mathrm{ji}}$ and also a weaker constraint on the connectivity $W_{\mathrm{ji}}$. In contrast, a larger penalty $C_{\mathrm{ji}}$ will push $W_{\mathrm{ji}}$ to approach 0.

For whole‐brain FC network construction, Equation (2) is rewritten as its equivalent matrix form:

(3) $$\min_{W}\frac{1}{2}∥X-\mathrm{XW}{∥}_{F}^{2}+\lambda ∥C⊙W{∥}_{1},$$

where $∥\cdot {∥}_{F}=\sqrt{\sum_{i,j=1}^{N}{\left(\cdot_{\mathrm{ij}}\right)}^{2}}$ is *F*‐norm of matrix, we denote $∥\cdot {∥}_{1=}^{\mathrm{def}}\sum_{i,j=1}^{N}\left|\cdot_{\mathrm{ij}}\right|$ in this work. We constrain $W_{\mathrm{ii}}=0$ to avoid the trivial solution W=I. To solve Equation (3), the alternating direction method of multiplier (Boyd et al., 2011) is adopted to calculate the connectivity network **W**. The detailed optimization process of Equation (3) can refer to (Yu et al., 2019). λ is a regularization parameter, related to the sparsity prior of WSR. Setting the sparsity prior λ with different values in range of $\left[2^{-4},2^{-3},\cdots ,2^{5}\right]$ can obtain d=10 sparse FCNs with different levels of sparsity, that is, $W^{\lambda_{1}},W^{\lambda_{2}},\cdots ,W^{\lambda_{k}}\cdots ,W^{\lambda_{d}}$.

According to Equation (3), WSR (penalizing the edges with different weights) is superior to SR which penalizes edges with a constant weight (i.e., 1). However, the edges corresponding to strong correlation are still penalized with the penalty weight, which makes the difference between strong and weak connectivity edges in the FC insignificant (i.e., the range of correlation is curtailed).

#### 2.3.3 The construction of sparsity‐guided multiple functional connectivity network

To characterize connectivity between paired ROIs influenced by the other ROIs, we propose to construct the sparsity‐guided multiple FCs based on PC and WSR. Specifically, we use PC to represent the connectivity strength, and WSR to represent the network sparse topology. Since the $W^{\lambda_{k}}$ constructed based on WSR is an asymmetric matrix, we adopt a strategy in Elhamifar and Vidal (2013) to symmetrize $W^{\lambda_{k}}$. Then, a binarization (0/1) operation is performed on $W^{\lambda_{k}}$, by which the non‐zero elements in $W^{\lambda_{k}}$ are set to 1, and the zero elements remain 0. The binarized format of $W^{\lambda_{k}}$ is denoted as $G^{\lambda_{k}}\in {\mathbb{R}}^{N\times N}$ and the sparsity‐guided multiple FCs are calculated by the following dot product operation on **P** and $G^{\lambda_{k}}$:

(4) $$M^{\lambda_{k}}=G^{\lambda_{k}}⊙P.$$

The $M^{\lambda_{k}}$ keeps strong linear correlation as in **P**, and alleviates the impact of the weakened connectivity strength caused by WSR. Applying $G^{\lambda_{k}}$ to **P**, the resulting FCN can both retain strong correlation for non‐zero interactions and eliminate false connections caused by the influence of other brain regions apart from the paired ROIs. By introducing a sparsity prior λ in WSR, the sparse FCN $M^{\lambda_{k}}$ can *not only* indicate the existence of crucial edges in **P**, *but also* remove the false connections effectively.

#### 2.3.4 Architecture of CNN

To explore the potential discriminative connectivity for SZ classification, the sparsity‐guided multiple FCs are used as the input of next step as shown in Figure 1b. Each sparse FCN corresponds to a two‐layer CNN, that is, FC‐Conv. For $M^{\lambda_{k}}$, each row/column in the FCN represents the correlation between a specific ROI and all the other ROIs. To find the latent high‐order spatial representation in the FCNs, we first employ a regional connectivity convolution kernel to learn the spatial mapping between a specific ROI and other ROIs by weighting and summing each row in the first layer. Specifically, we set the size of the regional connectivity convolution kernel as 1×N×H, where *H* is the number of channels, and then the output of the first layer is an N×1×H tensor.

To integrate the spatial mapping learned by the regional connectivity convolution, we introduce a spatial integration convolution kernel with the size of N×1×V in the second layer, and the output is the predictive feature with a reduced dimension learned from each FCN, that is, a 1×1×V flattened tensor. These predictive features from multiple branches are transformed into a $1\times 1\times \left(d\times V\right)$ vector, and input into two fully connected layers (with *D*_1_ and *D*_2_ neurons, respectively), and a softmax unit is used for SZ classification.

### 2.4 Implementation of SMFC‐net

The proposed architecture is implemented using python3 based on tensorflow2 and trained on a single GPU (NVIDIA GeForce RTX 3070) with 8GB of memory. In each FC‐Conv, the initialization of weights for two convolutional layers is “*he_normal*” (He et al., 2015), and the numbers of channels are set as H=64 and V=32, respectively. Each convolutional layer is followed by $l_{2}$‐norm of convolution kernel weight matrix ($l_{2}$ regular term coefficient is 0.00001). “Concatenation” and the first fully connected layer are followed by 0.2 dropout (Srivastava et al., 2014). The numbers of neurons in the two fully connected layers are $D_{1}=128$ and $D_{2}=64$, respectively. The number of neurons in the softmax layer is set to 2 for SZ classification. The Adam optimizer is used for training (Kingma & Ba, 2014), and the number of epochs, batch size and learning rate are empirically set as 200, 20, and 0.001, respectively. The SMFC‐Net is trained on the training data to minimize the cross‐entropy cost.

## 3 EXPERIMENTS AND RESULTS

### 3.1 Experimental settings

We employ the fivefold cross‐validation (CV) to evaluate our proposed method performance. Specifically, all subjects are randomly grouped into five subsets (each with equal size). Each subset is sequentially selected as a testing set, and the subjects in the remaining subsets are combined to construct the training set. In addition, we further randomly select 20% of the training set as the validation set for tuning the parameters, while the remaining 80% of the training set are combined to construct the internal training set. Nested fivefold CV is performed on the internal training set and validation set to select the optimal parameters that contribute to the best performance on the internal training set. This nested fivefold CV is repeated five times independently. The base classifiers for each fold in each repetition under the best parameter combination are kept for predicting the testing set. There are 5(fold)*5(repetition) = 25 base classifiers in total. The majority voting (i.e., whether a subject is positive or negative depends on the result predicted by the majority of the classifiers) is used to integrate the prediction results of these base classifiers to obtain the final prediction result. The above process is repeated 10 times independently for estimating the final performance.

We employ seven metrics to evaluate the performance of the method, including accuracy (ACC), sensitivity (SEN), specificity (SPE), balanced accuracy (BAC), receiver operating characteristic (ROC), area under the curve (AUC), and F_1_‐score (F_1_). The formulas of ACC, SEN, SPE, BAC, and F_1_ are as follows: $\mathrm{ACC}=\left(\mathrm{TP}+\mathrm{TN}\right)/\left(\mathrm{TP}+\mathrm{FN}+\mathrm{TN}+\mathrm{FP}\right)$, $\mathrm{SEN}=\mathrm{TP}/\left(\mathrm{TP}+\mathrm{FN}\right)$, $\mathrm{SPE}=\mathrm{TN}/\left(\mathrm{TN}+\mathrm{FP}\right)$, $\mathrm{BAC}=\left(\mathrm{SEN}+\mathrm{SPE}\right)/2$, $F_{1}=\left(2\times \mathrm{SEN}\times \mathrm{PPV}\right)/\left(\mathrm{SEN}+\mathrm{PPV}\right)$, where TP, TN, FP, and FN are true positive, true negative, false positive, and false negative, respectively. The positive predictive value (PPV) is defined by $\mathrm{PPV}=\mathrm{TP}/\left(\mathrm{TP}+\mathrm{FP}\right)$.

### 3.2 Method comparison

The proposed SMFC‐Net is compared with five conventional machine/deep learning methods, one advanced graph neural network (GNN) method as well as some variants of the SMFC‐Net.

*Support vector machine* (SVM) (Wee et al., 2011): The local clustering coefficient of each ROI is extracted from the matrix **P**. The vectorized local clustering coefficients, extracted from all nodes/ROIs, are then concatenated and fed into a linear SVM with default parameters for classification.

*PC‐CNN* (Bi et al., 2020): The matrix **P** is directly used as an input of the CNN. Specifically, the CNN contains three convolutional layers, two fully connected layers, and a softmax layer. Each fully connected layer is followed by 0.4 dropout. There is a max‐pooling layer behind each convolutional layer (pooling size = 2 × 2, strides = 2). The three convolutional layers have 16, 32, and 64 channels, respectively, and the corresponding filters have the same size of 3 × 3. The number of units in the two fully connected layers is 200, and the softmax layer is used for classification.

*WSR‐CNN* (Yu et al., 2017): The structure and parameters are same with PC‐CNN, only the input is replaced with $W^{\lambda_{1}}$.

*Long short‐term memory* (LSTM) (Dvornek et al., 2017): A recurrent neural network with long short‐term memory (LSTM) directly mines dynamic information from BOLD signals for SZ classification. For the subjects in the training phase, the BOLD signal was clipped into 10 sequences of length 14. We enlarged the training set by a factor of 10 by randomly varying the start time of each cropping sequence. The parameters follow the parameter settings indicated in Dvornek et al. (2017).

*Multilayer perceptron* (MLP) (Z. Wang et al., 2017): The upper triangular of **P** is extracted and converted into a 7140‐dimensional vector. The vectors of all subjects are standardized and used as the input of the MLP for classification. Specifically, the MLP consists of two fully connected layers (with 200 neurons) and a softmax layer. There is 0.4 dropout before and after the first fully connected layer.

*DiffPool* (Ying et al., 2018): DiffPool is a differentiable graph pooling module that can generate hierarchical representations of graphs and can be combined with GraphSage (Hamilton et al., 2017) based on message passing. Specifically, PC coefficients are used as node features and $M^{\lambda_{8}}$ as sparse graph. The graphs are separately fed into two DiffPool layers to learn feature representations, and then the features are concatenated and then classified using a fully connected layer. Each DiffPool layer contains two 2‐layer GraphSage, one of which is used to learn the cluster assignment of the pool, and the other is used to learn the pool node characteristics. The parameters follow the parameter settings indicated in Hu et al. (2021).

(*Hard Thresholding*) *HTFC‐Net*: To verify the effect of sparsity‐guided strategy on our method performance, HTFC‐Net is implemented without WSR sparsity‐guided PC, but employed a series of hard thresholds to directly construct multiple sparse FCNs from **P**, while the remaining network architecture is the same as that of SMFC‐Net. Specifically, based on the FC matrix **P**, edges with high absolute weights/values are retained by selecting thresholding parameters. The hard threshold parameter [10 20 30 40 50 60 70 80 90 99], indicates how many percent of the edges are discarded based on the absolute weights, and finally *d* FCNs with different sparsity are obtained.

(*Sparsity‐guided single*) *SSFC‐Net*: To verify the rationality of our multiple sparsity FCNs ($M^{\lambda_{1}},M^{\lambda_{2}},\ldots M^{\lambda_{d}}$), we separately use sparsity‐guided single FC matrix $M^{\lambda_{2}}$, $M^{\lambda_{4}}$, $M^{\lambda_{6}}$, $M^{\lambda_{8}}$, and $M^{\lambda_{10}}$, as input to a branch of SMFC‐Net, and other parameters remain unchanged. We name them as SSFC‐Net ($\lambda_{2}$), SSFC‐Net ($\lambda_{4}$), SSFC‐Net ($\lambda_{6}$), SSFC‐Net ($\lambda_{8}$), and SSFC‐Net ($\lambda_{10}$), respectively.

### 3.3 Classification performance

**FIGURE 2:**
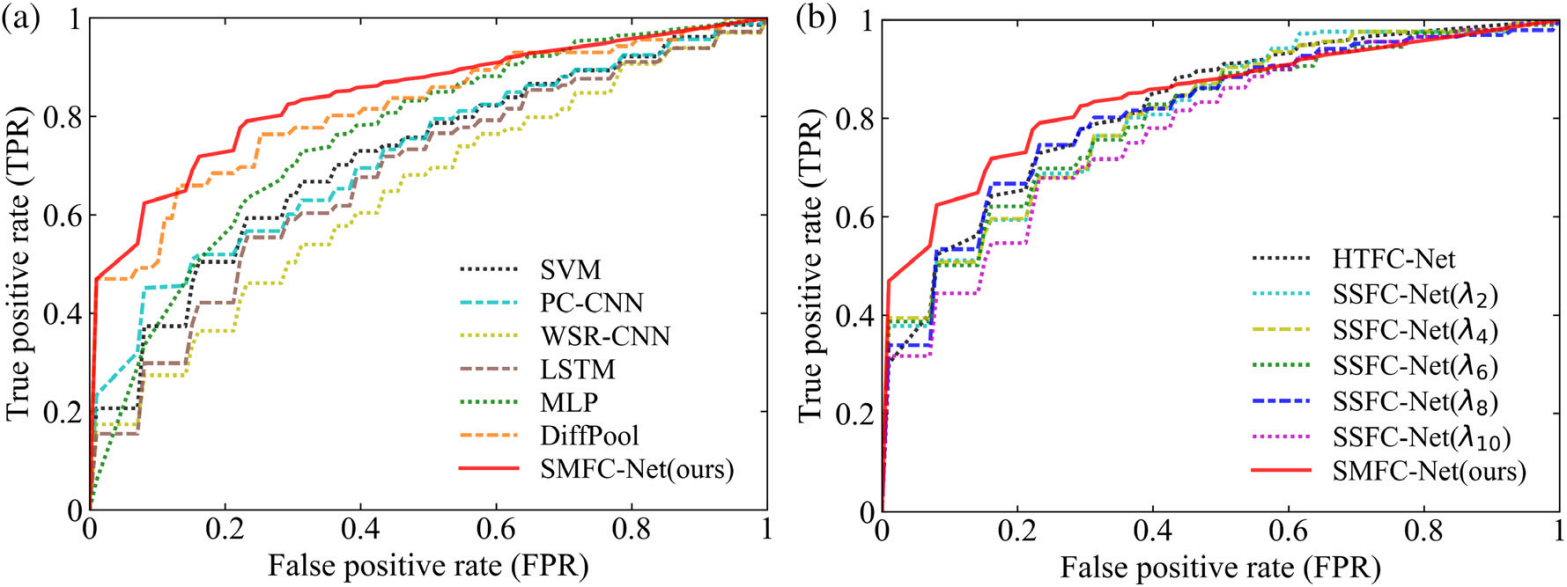
ROC curves produced by different methods. (a) Our method versus other competitive methods, and (b) Our method versus the variant methods. ROC, receiver operating characteristic.

The SZ classification performance using different methods is summarized in Tables 2 and 3, and the ROC curves are shown in Figure 2, where SZs are treated as the positive class.

**TABLE 2:** The comparison of performance between our method and other competitive methods in SZ classification (mean [std]%).

Method	ACC	BAC	SEN	SPE	AUC	F_1_
SVM	68.00 (1.43)	67.44 (1.96)	61.24 (1.49)	73.64 (2.42)	71.71 (1.40)	63.42 (1.36)
PC‐CNN	66.08 (3.00)	65.81 (4.84)	62.85 (3.76)	68.77 (5.92)	72.25 (2.46)	62.09 (2.88)
WSR‐CNN	59.84 (3.28)	58.47 (4.61)	43.65 (6.17)	73.29 (3.05)	64.29 (2.30)	48.31 (6.38)
LSTM	65.60 (2.40)	65.56 (5.05)	65.15 (5.47)	65.97 (4.63)	67.99 (1.66)	63.17 (3.17)
MLP	70.40 (2.82)	70.73(4.14)	74.18(3.92)	67.27(4.35)	75.82(2.39)	69.35(3.08)
DiffPool	72.00 (2.15)	72.26 (3.90)	66.42 (4.74)	78.10 (3.06)	81.44 (2.29)	67.51 (3.08)
SMFC‐Net (ours)	**78.16** (2.45)	**77.92** (3.18)	**74.97** (1.88)	**80.86** (4.47)	**84.43** (1.45)	**75.39** (2.01)

Abbreviations: ACC, accuracy; AUC, area under the curve; BAC, balanced accuracy; CNN, convolutional neural network; F_1_, F_1_‐score; LSTM, long short‐term memory; MLP, multilayer perceptron; PC, Pearson's correlation; SEN, sensitivity; SMFC‐Net, convolutional neural network with sparsity‐guided multiple functional connectivity; SPE, specificity; SVM, support vector machine; SZ, schizophrenia; WSR, weighted sparse representation.

**TABLE 3:** The comparison of performance between the proposed SMFC‐Net and variant methods in SZ classification (mean [std]%).

Method	ACC	BAC	SEN	SPE	AUC	F_1_
HTFC‐Net	71.84 (2.92)	71.61 (3.29)	68.21 (2.81)	75.01 (3.77)	82.23 (1.28)	68.31 (3.03)
SSFC‐Net $\left(\lambda_{2}\right)$	71.84 (2.70)	71.78 (3.45)	70.06 (4.39)	73.49 (2.50)	80.82 (1.74)	69.11 (3.56)
SSFC‐Net $\left(\lambda_{4}\right)$	72.16 (2.60)	71.82 (3.79)	68.64 (3.91)	74.99 (3.66)	80.82 (1.37)	69.00 (3.46)
SSFC‐Net $\left(\lambda_{6}\right)$	72.80 (1.24)	72.55 (3.60)	69.58 (4.30)	75.52 (2.90)	80.18 (1.45)	69.67 (2.34)
SSFC‐Net $\left(\lambda_{8}\right)$	74.56 (1.06)	74.23 (3.43)	70.61 (3.44)	77.85 (3.42)	81.04 (1.37)	71.24 (1.41)
SSFC‐Net $\left(\lambda_{10}\right)$	69.92 (1.65)	69.34 (3.29)	59.82 (2.98)	78.86 (3.60)	77.72 (1.92)	63.67 (2.40)
SMFC‐Net (ours)	**78.16** (2.45)	**77.92** (3.18)	**74.97** (1.88)	**80.86** (4.47)	**84.43** (1.45)	**75.39** (2.01)

Abbreviations: ACC, accuracy; AUC, area under the curve; BAC, balanced accuracy; F_1_, F_1_‐score; HTFC‐Net, convolutional neural network with hard thresholding functional connectivity; SEN, sensitivity; SMFC‐Net, convolutional neural network with sparsity‐guided multiple functional connectivity; SPE, specificity; SSFC‐Net, convolutional neural network with sparsity‐guided single functional connectivity; SZ, schizophrenia.

According to the results, one can have the following observations. *First*, the GNN method achieves better performance compared to five machine/deep learning methods (SVM, PC‐CNN, WSR‐CNN, LSTM, and MLP). For example, DiffPool achieves a 1.1% improvement in ACC values compared to MLP. This suggests that GNN methods may be more effective in brain disease identification. In particular, our proposed method achieves an accuracy of 78.16% for SZ classification. Excluding the variants of SMFC‐Net, the highest accuracy of other conventional methods is 72.00% (DiffPool). Compared to DiffPool, our method increases the accuracy by 6.16%, which proves that our method has more advantage in mining latent representations in the FCNs. In addition, PC‐CNN and WSR‐CNN have poor performance. This indicates that the FCN information is not hidden in the square neighborhood, which is consistent with the results of the existing research (Meszlényi et al., 2017). Compared with WSR‐CNN, PC‐CNN achieves relatively better performance, indicating that the weak connection had limited contribution to pattern recognition in deep learning. *Second*, compared with HTFC‐Net, the performance confirms that our proposed sparsity‐guided multiple FCs retain more discriminative FC, and eliminates more false connections in the FCNs. *Finally*, our SMFC‐Net achieves better performance than its variants, that is, SSFC‐Net ($\lambda_{2}$), SSFC‐Net ($\lambda_{4}$), SSFC‐Net ($\lambda_{6}$), SSFC‐Net ($\lambda_{8}$), and SSFC‐Net ($\lambda_{10}$), which implies that our strategy of employing multiple sparse learning can significantly contribute to the improvement of classification performance. Furthermore, SSFC‐Net ($\lambda_{8}$) achieves an accuracy of 74.56%, which is higher than SSFC‐Net ($\lambda_{2}$), SSFC‐Net ($\lambda_{4}$), SSFC‐Net ($\lambda_{6}$), and SSFC‐Net ($\lambda_{10}$). This shows that $G^{\lambda_{8}}$ ($\lambda =2^{3}$) leads to the lowest number of false connections and more reasonable brain network topology in the resulting FCNs.

### 3.4 Comparison with sota methods

We further compare our method with several SOTA studies using the COBRE data set. The results are listed in Table 4, where the data modality for all methods is fMRI, and the number of subjects used in different studies is reported. It is worth noting that these results are not fully comparable because different studies may have used different numbers of subjects. From Table 4, we can see that our proposed method achieves relatively competitive results compared to the SOTA studies. Our method is the first CNN‐based SZ diagnosis method utilizing spatial information in sparsity‐guided multiple FCs networks, while previous methods only focus on one aspect of these information or ignore the spatial information in FC networks.

**TABLE 4:** Comparison with state‐of‐the‐art methods in SZ diagnosis.

Method	Subject (SZ/HC)	ACC (%)	SEN (%)	SPE (%)
(Ji et al., 2019)	62/62	77.00	82.00	71.00
(Pan et al., 2022)	57/64	81.82	82.46	81.25
(Anderson & Cohen, 2013)	72/74	65.00	–	–
(Zeng et al., 2018)	71/74	73.60	68.00	76.60
(Huang et al., 2020a)	53/67	82.40	91.30	72.50
(Malkiel et al., 2021)	72/75	70.00	–	–
Our proposed	57/68	78.16	74.97	80.86

Abbreviations: ACC, accuracy; HC, healthy control; SEN, sensitivity; SPE, specificity; SZ, schizophrenia.

### 3.5 Important features in classification

#### 3.5.1 Important functional connectivities in classification

The occlusion method (Zeiler & Fergus, 2014) is applied to explore the important FC in SZ classification. We set the FC (i.e., element) existing in brain network (i.e., $M^{\lambda_{k}}$ matrix) to zero in turn, and send it to the trained SMFC‐Net model to get a new classification accuracy. By comparing the classification accuracy of SMFC‐Net, the importance of these FC is obtained according to the degradation of classification performance. Specifically, since $M^{\lambda_{k}}$ is symmetrical matrix, that is, $M_{\mathrm{ij}}^{\lambda_{k}}$ is equal to $M_{\mathrm{ji}}^{\lambda_{k}}$, we occlude two elements (i.e., the connections $M_{\mathrm{ij}}^{\lambda_{k}}$ and $M_{\mathrm{ji}}^{\lambda_{k}}$) of the input matrix $M^{\lambda_{k}}$ by replacing them with a zero‐occlusion mask. The zero‐occlusion mask moves in {$M^{\lambda_{k}}$} at the same time, and the occluded {$M^{\lambda_{k}}$} obtained by each movement is sent to the trained SMFC‐Net to obtain a new classification accuracy. $\left[N\times \left(N-1\right)\right]/2$ classification accuracies can be obtained, and they are subtracted from the accuracies of SMFC‐Net (78.16%) separately. The degradation of classification accuracies is treated as the contribution of each FC.

**FIGURE 3:**
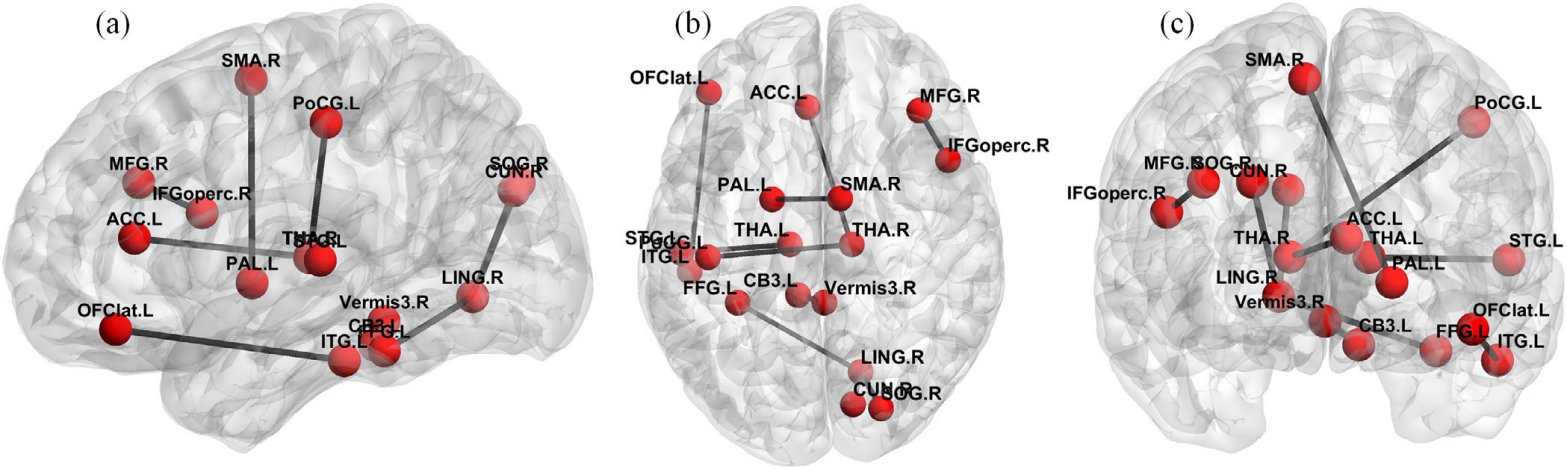
Top 10 important functional connectivity contributing the most in SZ classification. SZ, schizophrenia.

The top 10 important FCs in classification are shown in Figure 3. The names and abbreviations of the corresponding ROIs are listed in Table 5.

**TABLE 5:** The names, abbreviations, and indices of the ROIs corresponding to the top 10 functional connectivity that contribute most in SZ classification.

ROI index	ROI names	ROI abbr.
61 and 82	Postcentral gyrus left and thalamus right	PoCG.L and THA.R
52 and 54	Lingual gyrus right and superior occipital gyrus right	LING.R and SOG.R
16 and 79	Supplementary motor area right and pallidum left	SMA.R and PAL.L
31 and 93	Lateral orbital gyrus left and inferior temporal left	OFClat.L and ITG.L
52 and 59	Lingual gyrus right and fusiform gyrus left	LING.R and FFG.L
6 and 8	Middle frontal gyrus right and inferior frontal gyrus (opercular) right	MFG.R and IFGoperc.R
35 & 82	Anterior cingulate gyrus left and thalamus right	ACC.L and THA.R
81 & 85	Thalamus left and superior temporal gyrus left	THA.L and STG.L
50 & 52	Cuneus right and lingual gyrus right	CUN.R and LING.R
99 & 114	Left lobule III of cerebellar hemisphere and lobule III of vermis	CB3.L and Vermis3.R

Abbreviations: ROI, region of interest; SZ, schizophrenia.

From Figure 3 and Table 5, we can see that the top 10 connections with the highest contribution mainly involve the following ROIs, including the *Thalamus* (THA), *Supplementary motor area right* (SMA) right, *Lingual gyrus right* (LING) right, *Inferior frontal gyrus (opercular) right* (IFGoperc) right, *Lateral orbital gyrus* (OFClat) left, *Cuneus* (CUN) right, *Superior occipital gyrus* (SOG) right, *Fusiform gyrus* (FFG) left, *Inferior temporal gyrus* (ITG) left, and *Lobule III of vermis* (Vermis3.R). In previous studies (Andreasen et al., 1997; Li et al., 2012), these regions have also been reported to be highly correlated with SZ progression. Among these connections, SZ patients have greater thalamic connectivity with multiple sensory‐motor regions which is consistent with (Ferri et al., 2018). These results indicate that our method is effective in identifying SZ‐related FC.

#### 3.5.2 Important brain regions in classification

We also apply the occlusion method to identify the important brain regions for SZ classification. Specifically, we perturb all the connections with a specific ROI in the input matrix $M^{\lambda_{k}}$ by replacing them with an occlusion mask, typically a zero row/column vector. The mask moves across the matrices one ROI at a time. The degradation in accuracy is normalized to evaluate the contribution of each ROI. The contribution of brain regions in each independent experiment is as follows:

(5) $$D_{n}=\exp\left(\mathrm{AC}C^{\mathrm{our}}-\mathrm{AC}C_{n}^{\text{masked}}\right),$$

(6) $$C_{n}=\frac{D_{n}-\min{\left\{D_{n}\right\}}_{n=1,2,\cdots ,N}}{\max{\left\{D_{n}\right\}}_{n=1,2,\cdots ,N}-\min{\left\{D_{n}\right\}}_{n=1,2,\cdots ,N}},$$

where $D_{n}$ denotes the exponential form of degradation in accuracy, $n\in \left[1,2,\cdots ,N\right]$, $\mathrm{AC}C^{\mathrm{our}}$ denotes the ACC of SMFC‐Net, $\mathrm{AC}C^{\text{masked}}$ denotes the ACC obtained by feeding the occlusion masked input $M^{\lambda_{k}}$ into the trained SMFC‐Net, and $C_{n}$ denotes the normalized $D_{n}$ to indicate the contribution of the *n*th ROI.

**FIGURE 4:**
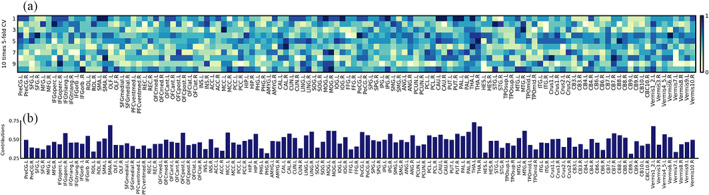
(a) The specific contributions of different ROIs in fivefold cross‐validation (CV) for 10 repetitive experiments. The vertical axis represents the number of CV, and the light/dark colors denote smaller/larger contribution of each ROI for classification. (b) The overall contributions of all cross‐validated ROIs in SZ classification. The vertical axis represents the overall contribution value. The horizontal axis represents the ROIs. ROI, region of interest; SZ, schizophrenia.

**FIGURE 5:**
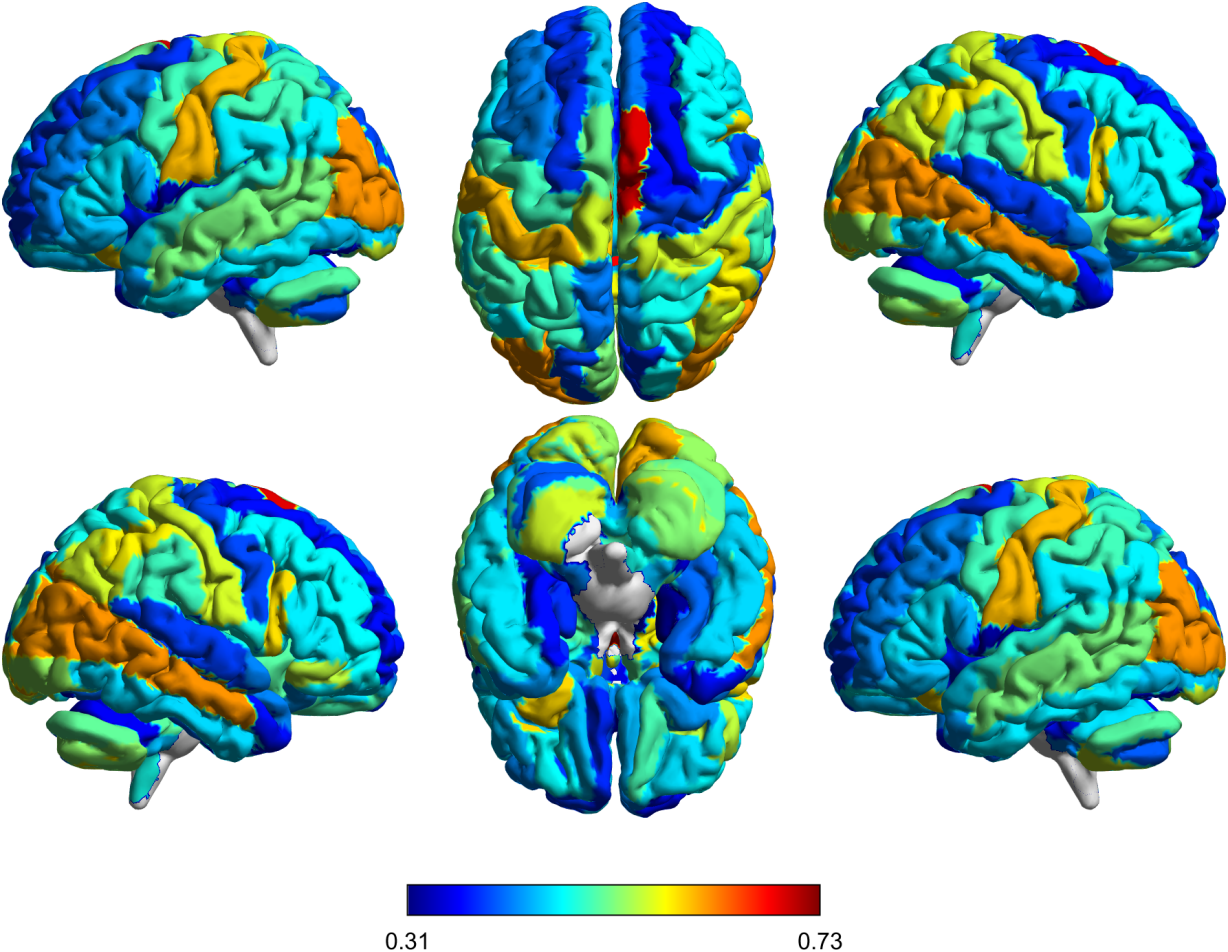
Visualization of contribution level of each brain region in the SZ classification. The color bar represents the contribution from small (blue) to large (red). SZ, schizophrenia.

Figure 4a demonstrates the results of fivefold CV for 10 repetitive experiments. The light colors indicate a lower contribution of each brain region to SZ classification, while the dark colors indicate a relatively larger contribution. Correspondingly, the overall contribution of ROIs under all experiments is calculated and demonstrated in Figure 4b. Furthermore, the overall contribution of the whole brain is visualized in Figure 5.

We can see from Figures 4 and 5 that several brain regions reveal their consistent importance among the multiple repetitive experiments, including the *Thalamus* (THA) left, *Supplementary motor area* (SMA) right, *Lobule I, II of vermis* (Vermis12), THA right, *Middle temporal gyrus* (MTG) right, *Middle occipital gyrus* (MOG) left, MOG right, *Lingual gyrus* (LING) right, *Pallidum* (PAL) right, and *Postcentral gyrus* (PoCG) left. The importance of these brain regions in SZ diagnosis has been reported in previous studies (Andreasen, 1997; Hoptman et al., 2010; Ichimiya et al., 2001; Onitsuka et al., 2004; Taylor et al., 2002; van Erp et al., 2016). Specifically, compared with HC, the SZ patients showed a decreased activation in SMA (Schröder et al., 1995), and a reduced gray matter density in both THA and PoCG (Glahn et al., 2008). The relatively high contribution values of these brain regions are also demonstrated in Figures 4b and 5.

## 4 DISCUSSION

Integrative analysis using multiple types of measurement for FC, such as PC and WSR, can take advantage of their complementary information and thus help construct sparse FCN containing more complex brain region interactions. In this paper, we propose sparsity‐guided multiple FCs, by integrating PC and WSR. This strategy *not only* retains the edge weights between paired ROIs, *but also* indicates the existence of edges in FCNs. This method can effectively and efficiently remove internal redundancy for constructing connectivity and generate multiple different sparse FCNs, which shows significantly advantage compared with previous studies that focus only on a single construction method (e.g., only using PC or WSR). Different from hard thresholding strategy, the sparsity‐guided method can more effectively and accurately eliminate false connections from the dense whole‐brain FCNs, by which FCNs with different levels of sparsity are generated.

For SZ diagnosis, we establish an SMFC‐Net framework to extract discriminative features from sparsity‐guided multiple FCs. SMFC‐Net can capture the high‐order spatial pattern, meanwhile considering the information complementarity of different sparsity FCNs. An occlusion strategy is applied to investigate the important FCs and brain regions for SZ classification, which can be regarded as a potential biomarker for identifying SZ‐associated aberrant connectivity. Interestingly, most of the connections listed in Table 5 focus on SZ‐pathology‐related brain regions that have been reported in previous studies as illustrated in previous sections.

To the best of our knowledge, our proposed method is the first attempt to integrate complementary information PC and WSR for constructing FCNs, and to build a CNN framework for analyzing those FCNs. The experimental results show that our proposed method outperforms several other FCN‐based methods, and demonstrates that our proposed method may provide important insights in revealing the underlying interactions of brain regions.

Although our proposed method has achieved great performance, it is still limited by the following several aspects. (1) The size of the data set uses in this study is relatively small, that is, only 125 subjects are used to evaluate the performance of the proposed method. Besides, the number of samples in different categories is also not balanced. In the future, we will evaluate our method on a larger and more balanced data set, that is, the autism spectrum disorder ASD data (Di Martino et al., 2014) from the Autism Brain Imaging Data Exchange data set. (2) Our work uses the static information in the BOLD signal to identify the disease. While, studies have reported that the dynamic information in the BOLD signals is conducive to improving the classification performance (M. Wang et al., 2019). Combining a variety of latent representations in BOLD to diagnose diseases will be our future work. (3) This work uses CNN architecture to identify the FC. The GNN has outstanding performance in recognizing patterns in FCNs (Huang et al., 2020b). In the future work, we will develop the framework based on the GNN to mine FCN, such that the classification performance can be potentially improved.

## 5 CONCLUSION

This paper introduces a novel SZ classification framework (SMFC‐Net) based on fMRI data. Our proposed method integrates PC which measures the statistical relationship between paired ROIs/nodes (*aka*, edge weight in FCN) and WSR which determines the existence of edges to construct sparsity‐guided multiple FCs. The FC‐Conv is applied to capture the high‐order joint spatial mapping between each central ROI and all other ROIs, and the learned embedding features from multiple structures of FCNs are combined for SZ classification. The experimental results from the COBRE database demonstrate the promising performance of our proposed method compared to the SOTA.

## Data Availability

This study uses publicly available data from the Center of Biomedical Research Excellence (COBRE). The detailed information of this data set can be found at http://fcon_1000.projects.nitrc.org/indi/retro/cobre.html.
